# Three millennia of heavy rainfalls in Western Mediterranean: frequency, seasonality and atmospheric drivers

**DOI:** 10.1038/srep38206

**Published:** 2016-12-02

**Authors:** J. P. Corella, B. L. Valero-Garcés, S. M. Vicente- Serrano, A. Brauer, G. Benito

**Affiliations:** 1 Institute of Physical Chemistry Rocasolano (CSIC), Madrid, Spain; 2 Instituto Pirenaico de Ecología (CSIC), Zaragoza, Spain; 3 Deutsches GeoForschungsZentrum Potsdam, Potsdam, Germany; 4 Museo Nacional de Ciencias Naturales, (CSIC), Madrid, Spain

## Abstract

Documenting subdecadal-scale heavy rainfall (HR) variability over several millennia can rarely be accomplished due to the paucity of high resolution, homogeneous and continuous proxy records. Here, using a unique, seasonally resolved lake record from southern Europe, we quantify temporal changes in extreme HR events for the last 2,800 years in this region and their correlation with negative phases of the Mediterranean Oscillation (MO). Notably, scarce HR dominated by a persistent positive MO mode characterizes the so-called Migration period (CE 370–670). Large hydroclimatic variability, particularly between CE 1012 and 1164, singles out the Medieval Climatic Anomaly, whereas more stationary HR conditions occurred between CE 1537 and 1805 coinciding with the Little Ice Age. This exceptional paleohydrological record highlights that the present-day trend towards strengthened hydrological deficit and less HR in the western Mediterranean is neither acute nor unusual in the context of Late Holocene hydrometeorological variability at centennial to decadal time scales.

One of the greatest challenges in climate science is understanding the effects of global warming on the spatial and temporal variability of heavy rainfall (HR) events and floods^1^. Changes in the frequency and severity of intense rainfall episodes directly impact the occurrence of floods, which in turn, affect many human and natural systems^2^,^3^. Therefore, it is essential to assess the long-term changes of extreme precipitation events to determine how climate variability processes and anthropogenic forcing influence these events^4^. Nevertheless, determining intensity, frequency and timing of extreme precipitation events is often difficult as currently available instrumental precipitation series are too short to identify significant changes at the tails of rainfall distributions.

Studies of past records based on geological (e.g. fluvial, marine and lake records) and historical data provide alternative means for characterizing past occurrences of extreme events and their response to climate variability. Flood layers embedded in annually laminated, or varved, lake records have been used to evaluate variability of extreme hydroclimatic events during the last centuries or even millennia^5^,^6^. Unfortunately, these records are very scarce and recent anthropogenic activities in lake watersheds often hamper calibrations of detrital layers against last century instrumental precipitation and/or water discharge observations^6^. Although there are several long varve sequences located in alpine environments, flood reconstructions may be biased towards spring and summer rainfall and/or snowfall melting^7^ as some lakes may freeze in winter. To understand the hydrological dynamics in the Mediterranean, where the main contribution to annual rainfall is cold season precipitation, records sensitive to rainfall extremes throughout the entire year are necessary.

The varved Lake Montcortès (Central Pyrenees) (Fig. 1) is an ideal study system as detrital layers are deposited after rainfall extremes throughout the year. The small size of the catchment (~1.4 km^2^) offers an excellent connectivity from the sediment source areas to the lake, avoiding temporal sediment sinks along the streams and slopes pathways. Furthermore, HR associated to detrital layers in this lake has been calibrated against instrumental climate data over the last 70 years^8^. All of the recorded HR events correspond to daily precipitation exceeding the 99^th^ percentile of all rainy days measured during the instrumental period (CE 1917–1994). Here we present a continuous 2,800-year-long record of storm-related runoff events that are indicative of changes in the HR frequency in the western Mediterranean, and demonstrate a robust link with atmospheric circulation modes as the Mediterranean Oscillation (MO). Particularly, we identify temporal patterns of “HR-rich” and “HR-poor” weather and illustrate changing rainfall seasonal patterns occurring during the Late Holocene. Moreover, using annual resolution and reconstructed seasonality of extreme precipitation events, we characterize the temporal stationarity of statistical parameters of extreme rainfalls in the light of well documented Late Holocene climatic periods in the North Atlantic region. Based on the relationship between flood layers, heavy rainfalls, and atmospheric circulation, the Lake Montcortés record demonstrate the decadal to centennial frequency variability of heavy rainfall affecting the western Mediterranean and the determinant influence of the Mediterranean Oscillation (MO).

## Results

### Recording seasonal indicators of heavy rainfalls within lake varve sequences

**Figure 1:**
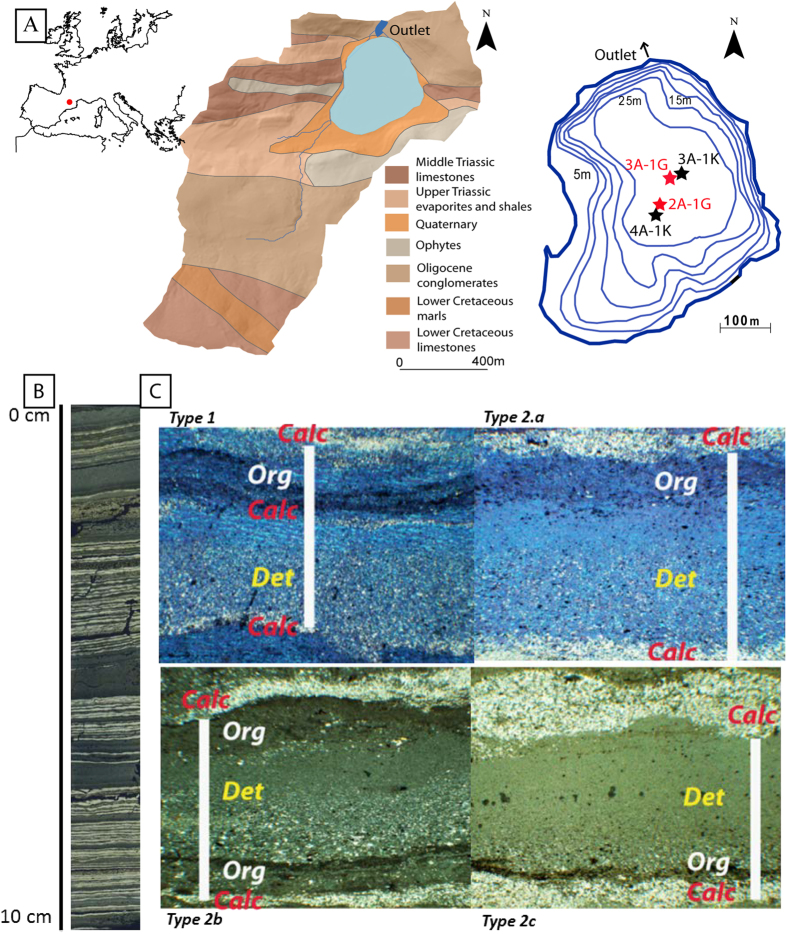
Location of Lake Montcortès and detrital microfacies within the biogenic varves. (**A**) Map showing the geological location of the lake watershed, a bathymetric map of Lake Montcortès and the location of coring sites (modified from Corella *et al*.^8^). (**B**) Scanned image of a thin section from Lake Montcortès’ sedimentary record showing couplets (black and white) of biogenic varves punctuated by detrital layers. (**C**) Polarized microscopy images showing the different microstratigraphic positions (seasonality) of detrital layers within the biogenic varve structure (white band). Type 1 (warm season), Type 2a (autumn), Type 2b (winter), Type 2c (early spring). *Geological map from* Fig. 1A *is reprinted from Quaternary Science Reviews (2014), Corella, J. P., Benito, G., Rodriguez-Lloveras, X., Brauer, A. & Valero-Garcés, B. L. Annually-resolved lake record of extreme hydro-meteorological events since AD 1347 in NE Iberian Peninsula, 93 Pages 77–90. Copyright (2014 Elsevier Ltd), with permission from Elsevier.*

Due to the meromictic conditions of Lake Moncortès, biogenic varves from the Late Holocene are well-preserved^9^,^10^ (Fig. 1B,C). Sediment cores retrieved from the distal basin of the lake (Fig. 1A) show biogenic annual varves composed of (i) endogenic calcite precipitated during summer (May-Sept) algal blooms and (ii) organic detritus (e.g. diatom frustules, amorphous organic matter and clay-sized material) deposited throughout the year^10^ (Fig. 1B,C). In addition, three types of detrital layers interbedded in the varve succession have been previously recognized^8^. *Non-continuous detrital layers (N-C DL)* consist of small clay-silt discontinuous layers, while *continuous detrital layers (DL)* are characterized by a normal grading from coarse-medium silt to clay with no micro-erosion features in the lower boundary. Both N-C DL and DL are deposited by low-density currents in the lake via inter- and overflows. The third layer type, *cm-thick flood-related turbidites (F-T)*, are related to high density underflow events and have a coarse basal sub-layer capped with a thinner clay-rich layer.

Detrital layers were deposited by runoff generated in the watershed during intense rainfall episodes. Our analysis focuses on the presence/absence of detrital layers to provide a comprehensive history of rainfalls above a minimum event threshold at seasonal resolution. During the instrumental period (CE 1917–1994), maximum daily precipitation (MDP) thresholds of 80 mm (R > 80 mm) recorded in Cabdella meteorological station were associated with deposition of N-C DL and rainfall over 90 mm (R > 90 mm) with DL and F-T^8^ (Table S1). The instrumental record also confirms that years with layers exceeding the MDP thresholds recorded a total annual precipitation 13–20% higher than average. Cabdella meteorological station is located ~13 km north of the lake and therefore different storminess magnitudes may have affected the two locations. Nevertheless, extreme precipitation events (>109MDP) recorded in the meteorological station have been always recorded in the lake sequence^8^.

The microstratigraphic position of detrital layers within biogenic varve couplets constrains the seasonality of flood layers^11^. Calcite precipitates in late spring and summer; therefore, detrital layers intercalated within calcite layers (lamination type 1) indicate warm season extreme rainfalls (May-Sept). In contrast, detrital layers embedded in organic layers (lamination type 2) correspond to cold season extreme rainfalls (Oct-April). Based on the position of the detrital layer before, within and/or after the organic layer (Type 2.a, b and c, respectively), we can infer if an extreme rainfall event occurred in autumn, winter or spring, respectively (Fig. 1C). Delayed warming in summer might bias the seasonality signal as calcite production may be affected; however, such a delay is rarely observed. Our analysis of detrital layers from the monitoring period (CE 1917–1994) shows approximately 82% of detrital layers are associated with cold season HR (i.e. lamination type 2), whereas only two layers are associated with warm season HR (i.e. Type 1 lamination), as confirmed by instrumental data collected at the nearby Cabdella meteorological station. This instrumental precipitation dataset shows that HR exceeding 80 and 90 mm occurred mainly during the cold season (Oct-April, 84.4% and 90% of the total events, respectively; Table S1); the remaining events correspond to summer rains. Therefore, accumulation of detrital layers in this lake is mainly associated with cold season meteorological phenomena. Lake sensitivity as a HR recorder has also been evaluated during the instrumental record: 30% of HR > 80 mm and 50% of HR >90 mm of the total cold season HR events are identified in the lake sequence (Table S1). These percentages suggest that Lake Montcortès paleohydrological archive may be biased towards lower efficiencies to record HR during the Late Holocene since HR may also correspond to local convective storms.

### Regional atmospheric circulation governing heavy rainfalls

Most HR recorded in Lake Montcortès occurred during the cold season (autumn-winter), which typically experiences cyclonic weather types and advective flows from the west (SW, W and NW directions; Table S1). Persistent rainfall episodes are commonly associated with negative sea level pressure anomalies over the Iberian Peninsula during dominant meridional circulation in the western Mediterranean (Fig. S2). The scarce summer HR events are caused by convective storms, a consequence of thermal related low pressure over the Iberian Peninsula^12^.

In the Iberian Peninsula, cold season extreme rainfall is therefore characterized by a low position of zonal circulation determined by the relative strengths of the main pressure systems in the Northern Hemisphere. Three important climate indices associated with north-south or east-west pressure oscillations have been defined for the western Mediterranean^13^,^14^, namely the North Atlantic Oscillation (NAO)^15^, Mediterranean Oscillation (MO)^16^,^17^ and Western Mediterranean Oscillation (WeMO)^18^. These three patterns, which explain large-scale atmospheric variability, are mainly active during the boreal winter, when they determine the climate over much of Europe^18^,^19^,^20^. In particular, the MO is characterized by an east-west pressure dipole across the Mediterranean and dominant meridional circulation that strongly influences precipitation variability in the entire Mediterranean region^16^,^20^.

**Figure 2:**
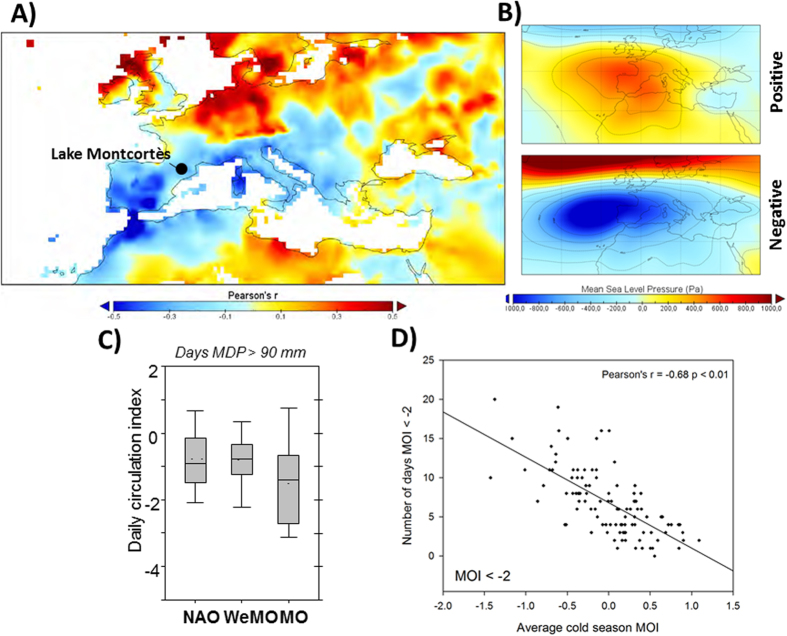
Correlation of the Mediterranean Oscillation with extreme precipitation in the western Mediterranean based on instrumental climate data. (**A**) Sea Level Pressure anomalies corresponding to the positive (MO > 1) and negative (MO > −1) phases of the MO (1901–2013). (**B**) October-April precipitation–MO index correlation for the period AD 1901–2013 using the CRU TS v. 3.22 data set. Figure 2A and B were generated using the Panoply 4.4.2 free software http://www.giss.nasa.gov/tools/panoply/ (**C**) Box plot showing the distribution of North Atlantic Oscillation (NAO), Western Mediterranean Oscillation (WeMO) and Mediterranean Oscillation (MO) daily values for HR events >90 mm, measured from Cabdella meteorological station between 1917 and 1994; (**D**) Relationship between the number of days in which the daily MO index was <−2 and the average MO index values during the cold season (October-April) for the period CE 1901–2013.

During the cold season, significant correlation between the (negative/positive) MO index and (high/low) total precipitation over the entire western Mediterranean region, including large areas of the Iberian Peninsula, France, Italy, Morocco and Algeria, is observed (Fig. 2A,B). This pattern is also observed at multi-decadal and multi- centennial time scales. The correlation between MO index reconstructions using surface pressure fields and independent reconstructions of precipitation for the period CE 1660–1999 clearly resemble the pattern obtained with instrumental data for the 20^th^ century (Fig. S3). This similarity further supports the general long-term stability and importance of the cold season MO in explaining rainfall variability in the western Mediterranean region.

Analyses of atmospheric circulation during instrumental HR (CE 1917–1994) around Lake Moncortès demonstrate that daily rainfalls over 90 mm during the cold season occurred during low negative MO modes. These results also suggest the MO index is showing a better predictive capacity compared to the NAO and WeMO indices (Fig. 2C). The frequency of days with a MO index less than −2, which usually resulted in heavy rainfalls in Moncortès (Table S1), is highly correlated with the average cold season MO index between 1901 and 2013 (Fig. 2D). Therefore, the record of heavy rainfall in Lake Montcortès is indicative of the prevailing atmospheric circulation controlling the seasonal MO in the western Mediterranean.

The sensitivity of Lake Moncortès to HR provides the basis for a 2,800-year-long record of changes in annual exceedance probability and frequency of extreme rainfalls. Changes in watershed sensitivity due to historic land use changes have been evaluated with vegetation dynamics proxies and geochemical indicators of run-off. Moreover, as main physical and limnological features of the lake have remained consistent during the late Holocene^9^, the flood layer record provides a sub-decadal reconstruction of the negative mode of the cold-season MO over the past three millennia.

### Late Holocene heavy rainfall variability reconstruction

**Figure 3:**
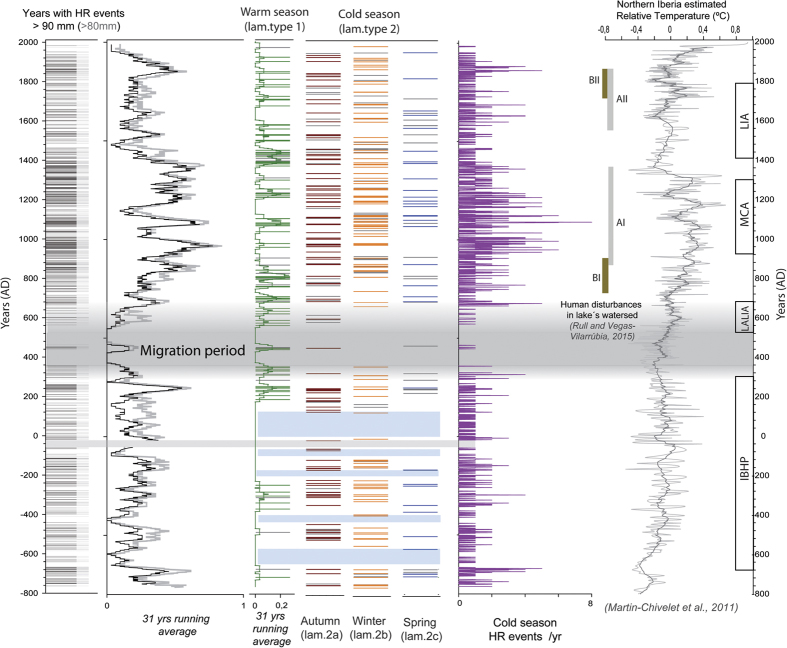
Extreme Heavy Rainfall events and seasonality reconstruction for the last 2,800 years. From left to right: Years with extreme precipitation events >80 mm (grey) and >90 mm (black) and seasonality based on microstratigraphic position of the detrital layers (see Fig. 1C). Pink boxes represent periods in which seasonality could not be clearly established. Total cold season HR events (without considering warm season HR events). Main anthropogenic phases derived from pollen data^24^. Estimate of relative land temperature change in northern Iberia based on δ13C analyses in speleothems^58^.

**Figure 4:**
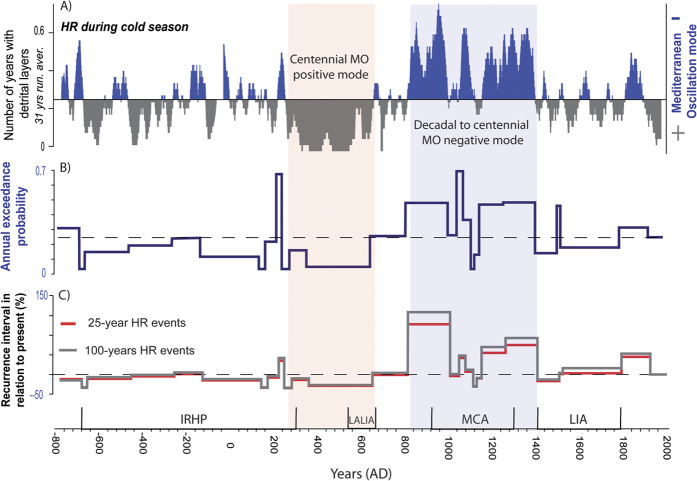
Late Holocene Mediterranean Oscillation modes and HR recurrence intervals. (**A**) 31-year running average of years with cold season HR detrital layers interpreted as a proxy of negative modes of MO (Note that positive MO modes are only represented by the lack of HR layers). (**B**) Changes in the annual exceedance probability of HR > 90 mm. (**C**) Variability of the recurrence intervals of the 25- and 100-year annual maximum daily rainfall events in relation to the present.

We identify a total of 1,220 HR > 80 mm layers (304 N-C DL, 700 DL and 216 F-T) distributed over 819 of the 2,775 years examined (BCE 763- CE 2012). Multiple HR events are identified for 245 years. Strikingly, the period CE 927 to 1398 alone recorded 118 years with multiple HR layers occurring mainly during the cold season (up to 5 HR events/yr; Fig. 3). HR > 90 mm layers are recorded in 916 cases (over 621 years). Rainfall seasonality was determined for 422 years, comprising 109 warm (lamination type 1) and 313 cold (lamination type 2a, b and c) season HR layers. Both the total number of detrital layers and their seasonal distribution fluctuate greatly over the past 2,775 years, as summarized in Fig. 3. A statistical treatment of the dataset has been applied to the HR > 90 mm events since they are recorded more frequently in the lake providing a more robust reconstruction of extreme events in the area. These statistical analyses of the mean time between events exceeding the HR > 90 mm threshold (Fig. S5) highlight periods with stationary regimes (mean and variance), which are a result of more stable hydroclimatic conditions in the region (Fig. 4B). Our analysis shows that major differences in the frequency of extreme events in the Montcortès lake record are consistent with main climate periods described for the North Atlantic region, with higher hydroclimatic variability observed during transitional periods (Figs 3 and 4).

*The Iron Age-Roman humid Period (IRHP; BCE 650-CE 300).* Heavy rainfalls during the IRHP mostly occurred during the cold season, likely due to negative MO modes. Five major stationary phases (each 100-270 years in duration) are recognized (Fig. 4B). The HR recurrence rate before the onset of the IRHP (BCE 765-670) was higher than the present-day rate. During the late Iron Age (BCE 640-115; Iberian Period), we observe an increasing trend in average annual HR occurrence that significantly dropped at the time of the Roman conquest of Hispania around BCE 135. The Roman Empire coincided with a 270-year-long period (BCE 113-CE 155) of stationary conditions. However, the average HR frequency observed for that time is about half that of the present time (CE 1938-1993). At the end of the IRHP, highly irregular extreme weather patterns are observed, characterized by alternating multi-decadal periods with absence of detrital layers and HR-rich episodes with an average frequency three times higher than current rates. Most of this period (BCE 520-CE 248) shows marked seasonality with a large increase in autumn HR (lamination Type 2a) and few warm season layers observed until CE 188.

*The Migration Period and the Late Antique Little Ice Age (LALIA; CE 300-670).* An exceptional period with a very low frequency of cold season HR occurred in Lake Montcortès between CE 370 and 670, particularly until CE 575. During this 300-year period, only two HR events occurred (autumn of 453 CE and spring of 464 CE), indicating a persistent positive-like mode of MO over centennial time scales (Fig. 4A). A reduction in extreme precipitation events also occurred during the warm season over a period of ~200 years (between CE 354 and 563). Frequency analysis shows that HR layers occurred at 25-year recurrence intervals, 10 times longer than current intervals, which occur, on average, every 2-3 years (Fig. 4B,C).

*The Medieval Climate Anomaly (MCA; CE 900 to 1300).* A shift towards dominant negative MO values occurred during the LALIA/MCA transition as reflected by the progressive increase in cold season detrital layers (Fig. 4A). The LALIA/MCA transition was also marked by an abrupt increase in warm season HR at CE 681, lasting until CE 845 (Fig. 3). The MCA highlights a strong hydrological contrast with a negative MO mode operating during two phases of increased cold season HR (mostly autumn and winter events) at CE 829-1012 (HR > 90 mm twice more frequent than than current rates) and CE 1061-1090 (HR > 90 mm three times more frequent than current rates). A marked seasonality also occurred during the MCA with reduced spring and warm season rainfall events between CE 916 and 1070, and a complete absence of the latter between CE 1106 and 1213 (Fig. 3).

*The Little Ice Age (LIA; CE 1300–1850) and the Industrial Period (after CE 1850).* The MCA-LIA transition represents an exceptional 250-year period with abundant cold season HR, indicating a persistent negative MO mode between CE 1164 and 1414 (2- year recurrence intervals for HR > 90 mm). The onset of the LIA is also characterized by the largest increase in warm season HR between CE 1372 and 1452. The second part of the LIA is punctuated by two periods, CE 1508–1547 and CE 1592–1656 with negative MO conditions and a relatively higher frequency of cold season HR. Based on the total number of HR events during this time-interval, a period of 270 years (CE 1537–1805) shows stationary conditions with a relative low HR frequency (5.4 years average recurrence) compared to the Industrial period (4 years average recurrence). The Industrial period is characterized by an increase in cold season HR and negative MO conditions between CE 1845 and 1900 and by an overall reduction of extreme events during the 20^th^ century, particularly since the 1950s.

## Discussion

### Influence of land use changes on the sediment delivery to the lake

Land use changes such as forest clearing and agriculture intensification may increase erodibility in the lake´s catchment, altering the sediment dynamics in the watershed. Thus, an increase of grazing activities and farming may enhance soil erodibility as observed in similar karstic systems^21^. Several studies in other lacustrine sites have used physical characteristics of detrital layers, such as thickness or particle size, to infer palaeoflood magnitudes^22^. Previous studies in Lake Montcortès have demonstrated historical changes of land use and periods of higher human disturbance in the catchment^9^,^10^,^23^,^24^, which may obscure further interpretation of flood magnitudes. For this reason, we solely use occurrence of detrital layers (and not the thickness) to document HR in the region since the anthropogenic imprint would not influence the timing of these events. The comparison of 20^th^ century instrumental climate data from Lake Montcortès‘ nearby meteorological stations shows a good matching of detrital layers deposition at years of occurrence of extreme heavy rainfalls. The agreement between Lake Montcortès HR frequency reconstruction with the historical floods record beyond the instrumental period^8^ in NE Spain and W Mediterranean supports the climatic origin of these detrital layers.

However, it is important to note that these MDP thresholds might have varied in the past under different land use conditions, and that it could be expected an increased in the system´s ability to record HR < 80 mm events during periods of more intense land use in the area. In order to discern the main controls affecting run-off generation, the influence of these human activities in Lake Montcortès should be fully evaluated as the anthropogenic pressure in the watershed may have induced an increased flood-sediment accumulation (in terms of volume) in the sediment record. Documentary and palynological records point to reduced anthropogenic disturbances in the area before and during the Migration Period (Fig. S4) suggesting absence of HR as the main cause for the lack of detrital layers in Lake Montcortès during this period. A palynological study from Lake Montcortès extending back to ~700 AD^24^ showed two periods of intensified vegetation burning (BI and BII^24^) occurring between 8-9^th^ and 17^th^ and mid- 18^th^ centuries, respectively. These burning phases may have had a considerable influence in the sediment yield in the watershed, mobilizing large amounts of sediment to the lake. Nevertheless, the frequency of detrital layers shows low sensitivity to the fire activity in the area (Fig. 3), as BI and BII coincide with periods of reduced detrital layer deposition. The vegetation evolution in Lake Montcortès^23,24^ also showed two major periods of agricultural development between 9–14^th^ and 16–19^th^ centuries, phases AI and AII respectively (Fig. 3)^24^. Human disturbances in the lake watershed during AI phase (i.e. Middle Ages) most likely increased the sediment delivery to the lake, and thus, the increase of detrital layers frequency during the Middle Ages may respond to the interplay between human activities and increased storminess. Contrarily, a major agricultural expansion in the area between the 16–19^th^ centuries^24^ does not show a synchronous pattern with anomalous higher number of detrital layers (Fig. 3). In addition, a regional increase in cultivated areas between CE 1470–1850 documented in a nearby Pre-Pyrenean lake^25^,^26^ (Fig. S4) does not coincide with any increase in the frequency of detrital layers in Lake Montcortès. Although historical periods of higher human disturbance may have increased overall sediment delivery from the catchment into Lake Montcortès, data suggest that climate rather than land-use change has been the major driver controlling the frequency of detrital layers in the lake.

### Paleoclimatic implications of Lake Montcortès paleohydrological record

Varve preservation in Lake Montcortès began at BCE 763. This period coincides with the end of global cool and dry conditions between 3.4 and 2.7 cal ka BP^27^, particularly well documented in the western Mediterranean^28^. Lake Montcortès’ varved record has shown how climate variability controlled the frequency and seasonality of HR, which in turn affected the mean recurrence interval (stationarity) of extreme rainfall over time. The driving factors causing departures from stationarity reveal changes in North Atlantic atmospheric circulation patterns. Specifically, the cold season HR variability observed in Montcortès indicates changes in low frequency, cold season MO patterns and reveals the hydrological impact of these changes over the western Mediterranean at multi-decadal time scales.

An increase in cold season HR, suggesting a persistent negative MO mode, occurred during the late IRHP period. This increase likely resulted in greater water availability in the Iberian Peninsula during the Iberian and Roman period with a concomitant reduction in summer precipitation. Our results corroborate a previous hypothesis suggesting wetter winters with drier summers in the Ebro basin between 900–300 BCE^29^ and a large increase is autumn precipitation between BCE 400 and CE 100 in Western Mediterranean, based on ^13^C analyses in fossil charcoal remains^30^.

A conspicuous period with scarce HR (CE 370–670) occurred during the so-called Migration period and may be related to a long persistent positive-like MO mode. The direct relationship between HR and total annual precipitation indicates a long-lasting hydrological deficit in the western Mediterranean during the Migration period. The absence of palaeoflood records in fluvial archives from western Mediterranean rivers^31^ and the reduced flood frequency and lower lake levels in southern and western Alps^6^,^32^,^33^ further support this hypothesis. Interestingly, during the same period, the most intense and severe cold season precipitation episodes occurred in Central Turkey^34^. This synchronous see-saw pattern of abrupt climate shifts was generally in western and eastern Mediterranean regions, suggest a common climate driver. Our data also suggest a link between less frequent cold season HR and dominant high sea level pressures over the western Mediterranean, both characteristic of positive MO phases. Lower summer temperatures experienced by central Europe and the western Mediterranean at that time^35^, particularly during the LALIA (CE 536–660)^36^, would contribute to less frequent summer thermal low pressure conditions, thereby resulting in less convective storms. The socioeconomic impacts of these climate changes (e.g. a hydrological deficit coupled with cold temperatures) likely significantly affected agricultural-based societies of Rome and northern Europe^36^. Interestingly, these unprecedented 330 years with scarce HR and long-lasting positive MO modes correspond to the collapse of the Roman Empire and the Germanic invasions. The prolonged aridity crises led to desiccation of lakes in central Spain and a subsequent decline of nearby human settlements^37^.

The shift towards a high variability of MO values occurred during the LALIA/MCA transition and several phases within the MCA were characterized by negative MO values (i.e. CE 829–1012, 1061–1090 and 1164–1414) (Fig. 4A). The interplay between more intense human activities and a high hydroclimatic variability - particularly between CE 1012 and 1164 with alternating decades of HR-poor and HR-rich conditions - explain the increase in detrital layers that characterizes the sedimentary record during the MCA (Figs 3 and 4). During the MCA, 25% of the years had multiple HR events, indicative of frequent occurrence of cold-season cut-off-low atmospheric processes. Although most available records indicate lower lake levels in the Iberian Peninsula during the MCA^38^, there is not a lineal relationship between decreased water availability and HR events. Most of the paleohydrological reconstructions based on lake records from NE Iberian Peninsula represent long-term changes in effective moisture and hydrological balance, which does not necessary have a direct link to the occurrence of extreme events (i.e higher storminess may have occur under humid or drier periods indistinctively). In addition, our study shows a strong hydrological contrast with increase cold season precipitation during two periods at CE 829–1012 and CE 1061–1090, but also reduced precipitation during other intervals within the MCA. The decrease in warm season HR during the 10^th^ and 12^th^ centuries combined with the warmer conditions that occurred during the MCA would have led to a strong summer hydrological deficit that may explain the lower lake levels recorded in the Mediterranean fringe of the Iberian Peninsula during these periods^38^. The high variability of the hydroclimatic signal preserved in Lake Montcortés during the MCA is in agreement with lake records in Western Alps^39^ and with fluvial and documentary archives in western Mediterranean, showing higher flood frequencies, particularly intense during CE 1000–1150^31^,^40^. Lake Montcortès sediments also recorded persistent negative MO modes during some phases of the LIA and the Industrial period (e.g. CE 1508–1547, 1592–1656 and 1844–1902).

A high frequency of warm season HR (and rainfall variance alternating HR-poor and HR-rich decades) are clustered in three main periods at AD 188–354, AD 681–845 and AD 1372–1452 with frequencies of 0.08, 0.12 and 0.17 HR events/yr respectively. These frequencies are two to four times higher than the average warm season HR frequency for the studied interval (0.04 HR events/yr) and occurred during transitional period between main climate phases (i.e. end of the IRHP, LALIA-MCA and MCA- LIA, Fig. 3). This fact supports the hypothesis of increasing flood frequencies during transitional climatic periods^41^.

The current HR frequency is not exceptional in the context of the last 2,800 years and has, in fact, decreased since the 19^th^ century. Future projections for the western Mediterranean show that, for a 20-year return period (CE 2081–2100), HR maximum daily precipitation will increase in frequency about 15–20%^42^. However, these projections are highly uncertain and some are even contradictory^43^. The lake Montcortès record provides analogues for HR frequency distributions for future warmer climate conditions. Thus, this record shows that more frequent heavy rainfall events have occurred during warmer climate periods (e.g. MCA) than in other periods with cold conditions such as the LIA (Fig. 3). The MCA also shows the largest hydrometeorological variability: the magnitude of the 25 year daily precipitation maximum exceeded, by more than 50%, the ones recorded during the instrumental period CE 1917–1994 (Fig. 4C). As a result, analysis of the three millennia heavy rainfall record from Lake Montcortès suggests that the frequency of extreme hydrological events is likely to increase under continued future warming conditions.

## Methods

### Study site

Lake Montcortès (maximum water depth ~30 m, ~1.4 km^2^ surface area; Fig. 1A) is located in the southern Pre-Pyrenees at 1031 m a.s.l. It was formed by karstic processes of dissolution and collapse on Triassic evaporites^9^. The lake’s watershed is emplaced on Oligocene conglomerates and siliciclastic, carbonate and evaporitic Mesozoic rock formations with some hypovolcanic ophite bodies. There is no permanent streamflow inlet to the lake, and the lake level is controlled by an outlet stream located along the northern shore (Fig. 1A).

### Coring and microfacies description

Long (MON04-3A-1K and MON04-4A-1K) and short (MON12-3A-12G and MON12-2A-12G) sediment cores were retrieved from the distal basin with a Kullenberg coring platform in 2004 and an UWITEC gravity corer in 2012, respectively. Sediment cores were split lengthwise and correlated by visual description and geophysical (Magnetic Susceptibility) and geochemical (X-ray Fluorescence) profiles. A composite sequence of 543.5 cm was sampled for large-scale thin sections (120 mm × 35 mm) for the identification of the different sedimentary microfacies and varve counting.

### Age model

An independent absolute varve chronology was obtained from the complete varve sequence in Lake Montcortès (543.5 cm) by analyzing the composite sedimentary sequence from CE 2012 to BCE 763 (Fig. S5). Core correlation was assessed by detailed inspections and varve counting of thin sections. Mean counting error ranges between 0 and 3%, depending on varve quality at different depths^10^. Varve interpolation was needed in 12.6% of cases due to poor varve preservation. These intervals were interpolated by using the mean varve thickness of the upper and lower centimeters of these intervals. Varves during the period CE 2012-118 (last 1,894 years) are very well preserved and only 1.2% of these varves required interpolation. In contrast, the period CE 117- BCE 762 displayed more frequent intervals with poor varve preservation (37% of cases). The interval BCE 19–53 (core depth 496–498 cm; grey band in Fig. 3) corresponds to a stratigraphic area where the sediment was disturbed. Varve chronology is in agreement with two independent dating methods— the AMS ^14^C age model ^10^ and ^210^Pb chronology for the recent sediments^8^ (Fig. S5)—both of which support the robustness of the varve chronology. The allocthonous input to the lake results in significant changes in the sediment accumulation rate (SR) in the lake. Two periods, CE 1901-1852 and CE 1362-563, had high sediment delivery with SRs of 0.69 and 0.45 cm/yr, respectively; the periods CE 2012-1902, CE 1851-1363 and CE 562-BCE 762 display SRs of 0.12, 0.11 and 0.6 cm/yr, respectively.

### Instrumental climate data in the lake

Daily precipitation instrumental data, available since CE 1917, was obtained from Cabdella meteorological station, located 15.6 km north of Lake Moncortès (Table S1, Fig. S1). Daily precipitation data underwent careful quality control and homogenization using a percentile-based rule with other meteorological stations from the SE Pyrenees. Homogenization of the series was tested using the relative standard normal homogeneity test (SNHT) developed by Alexandersson (1986)^44^ for single breaks based on a reference series created from neighboring meteorological stations.

### Atmospheric pressure data and weather-types classification

To determine the weather types generating high precipitation events around Lake Moncortès (Table S1), we utilized a common method formulated by Jenkinson and Collison (1977)^45^ based on the Lamb catalogue^46^. To apply this methodology, a sea-surface pressure grid of 16 points centered over Great Britain and Denmark, obtained by Jones *et al*.^47^, was transposed over the Iberian Peninsula. Wind types and directions and their vorticity in geostrophic units (hPa) were calculated from daily pressure data at these points to obtain a classification of weather types for the period CE 1915–2008. We used the NCEP-NCAR daily surface pressure data set (ds010.0) for this analysis (http://rda.ucar.edu/datasets/ds010.0/)^48^,^49^ with a spatial coverage of 5 × 5 degrees. The spatial patterns of atmospheric circulation configurations for high precipitation days in which detrital layers were formed (Fig. S2) were obtained from the 20^th^ Century Reanalysis^50^, thus having higher spatial resolution (2º × 2º) than the ds010.0 dataset. Long-term averages for Sea Level Pressure (SLP) were calculated using daily SLP anomalies.

### Atmospheric circulation indices

SLP grids from the NCEP-NCAR daily surface pressure data set between 1901 and 2013 were used to calculate NAO, MO and WeMO daily indices. Indices were calculated as the daily normalized difference between the SLP at the dipoles 35N, 5W and 65N, 20W (NAO); 35N, 5W and 30N, 35E (MO) and 35N, 5W and 45N, 10E (WeMO). A cold-season (October-April) MO was calculated averaging the monthly values. The spatial configurations of the cold season positive and negative MO phases were obtained averaging the SLP anomalies over the SLP grid points (obtained from the ds010.0 dataset) over the entire Mediterranean region corresponding to the years that showed a cold-season MO > 1 for positive years and a MO < −1 for negative years. The spatial configuration of the influence of the cold- season MO on the Mediterranean climate was assessed correlating the cold-season MO from 1901 to 2013 with precipitation from the Climate Research Unit (CRU) TS v. 3.22 data set^51^.

### Paleoclimatic reconstructions

We used the paleoclimatic database developed by Pauling *et al*.^52^, which provides precipitation reconstructions since CE 1500 at the seasonal scale. The SLP for the winter season of the region 70N–30W, 30N–40E (available for the period CE 1659–1999^53^) were also used. The predictors used to reconstruct SLPs were independent from those used for the precipitation reconstructions — for details, see Pauling *et al*.^52^. A winter MO index was obtained from 1659 to 1999 using the closest grid points to Gibraltar and Lod, as described above. Correlation between the winter MO and winter precipitation fields from Pauling *et al*.^52^ was calculated.

### Flood frequency analysis

The timing and characteristics of detrital layers overlapping the recorded instrumental rainfall period were compared with the series of maximum daily precipitation (Table S1). The continuous detrital layers and turbidite facies were associated with daily rainfall events exceeding 90 mm, representing a peak over threshold record of HR covering the last three millennia. A statistical test^54^ to detect trends and changes in the stationarity of the cumulative number of over-threshold values (m_t_) that occurred within an interval [0; t] was applied (Fig. S6). The null hypothesis H_0_ assumes the process of occurrence of these floods can be described by a homogeneous Poisson process:


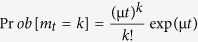


With expected number of over-threshold events by time t equals E [mt] = μt, and 1/μ is the mean time between events exceeding a threshold R > 90 mm. The rejection of this test is verified when the cumulative number of floods (NS) over the interval [0; t_end_] is outside the 90% tolerance interval (95% and 5% quantiles) which implies the failure to comply with the Poisson process hypothesis (non-independence and non-homogeneous datasets). The series stationary test was applied to the Lake Montcortés HR > 90 mm events between 763 BC and AD 1993, which consist of a total of 642 exceedances. The null hypothesis H_0_ was rejected when at least 20% of the points fell outside the range of a 10% significant level. This analysis identified 29 periods having stationary characteristics related to the occurrence of storms. It also identified break points highlighting periods with different durations and frequencies. The duration of stationary periods varied between 291 (AD 373–664) and 15 (AD 1520–1536) years; the mean time (Tr) between detrital records ranged from 1.43 to 18.19 years. The annual exceedance probability of R < 90 mm corresponds to the inverse of average timing between records (1/Tr).

Flood frequency analysis (FFA) was performed for each period with statistical stationarity. A set of probability distribution functions (TCEV, GEV, SQRT-ETmax) was fitted to the reconstructed flood data series and the parameters of these distribution functions were estimated by the maximum likelihood method^55^. This method was selected based on its statistical features performance for large samples and for its capacity to easily incorporate in the estimation process any additional non-systematic quantified data^55^,^56^. The square-root exponential type distribution of the maximum (SQRT-ETmax) function^57^ was finally selected on the basis of best statistical estimators of adjustment. The maximum daily precipitation with average recurrence intervals of 25 and 100 years were calculated for each identified period with stationary characteristics of the exceedance HR events (Fig. S6).

## Additional Information

**How to cite this article**: Corella, J. P. *et al*. Three millennia of heavy rainfalls in Western Mediterranean: frequency, seasonality and atmospheric drivers. *Sci. Rep.* **6**, 38206; doi: 10.1038/srep38206 (2016).

**Publisher's note:** Springer Nature remains neutral with regard to jurisdictional claims in published maps and institutional affiliations.

## Supplementary Material

Supplementary Information
