# Supplementary material for: Three millennia of heavy rainfalls in Western Mediterranean: frequency, seasonality and atmospheric drivers
Source: Sci Rep. 2016 Dec 2;6:38206. doi: 10.1038/srep38206 (PMC5133600; doi:10.1038/srep38206)
Supplement: Supplementary Information [file srep38206-s1.pdf]

Three millennia of heavy rainfalls in Western Mediterranean: frequency, seasonality and atmospheric drivers

J.P.Corella, B.L. Valero-Garcés, S.M. Vicente- Serrano, A. Brauer and G. Benito

Supplementary information

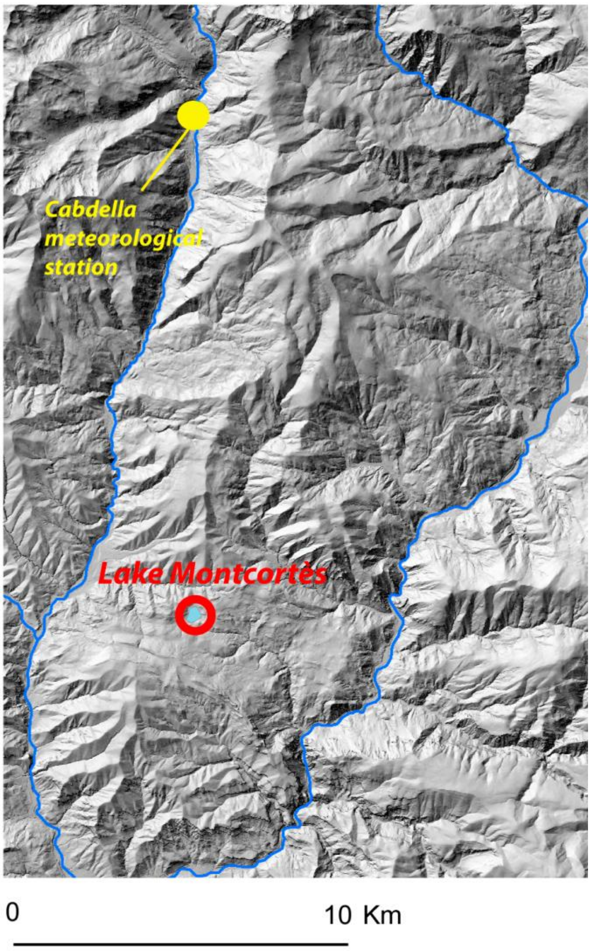

Figure S1: Digital Terrain Model (5 m resolution) of the Pallars Sobira (NE Spain) region showing the location of Lake Montcortès and Cabdella meteorological station

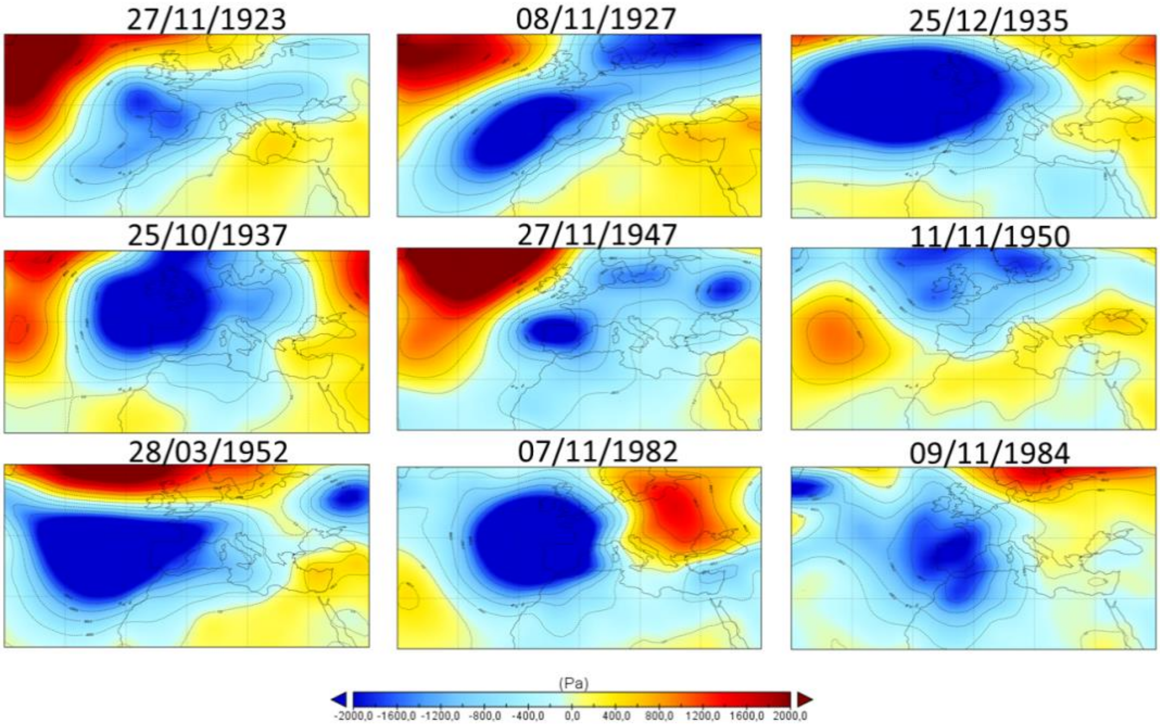

Figure S2: Sea Level Pressure (SLP) Anomalies during the cold-season extreme rainfall events that triggered detrital layers recorded in Lake Montcortès since CE 1917. Maps were generated using the Panoply 4.4.2 free software (http://www.giss.nasa.gov/tools/panoply/)

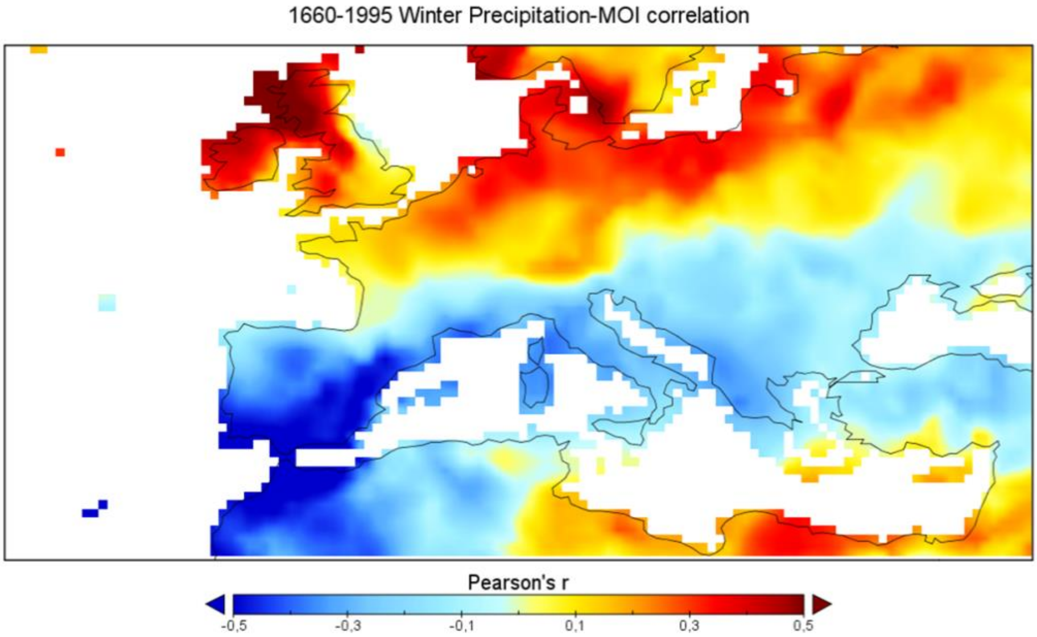

Figure S3: CE 1660-1999 Winter Precipitation – MOi correlation based on gridded data from paleoclimatic reconstructions of precipitation [1] and Sea Level Pressure [2]. Map was generated using the Panoply 4.4.2 free software (http://www.giss.nasa.gov/tools/panoply/)

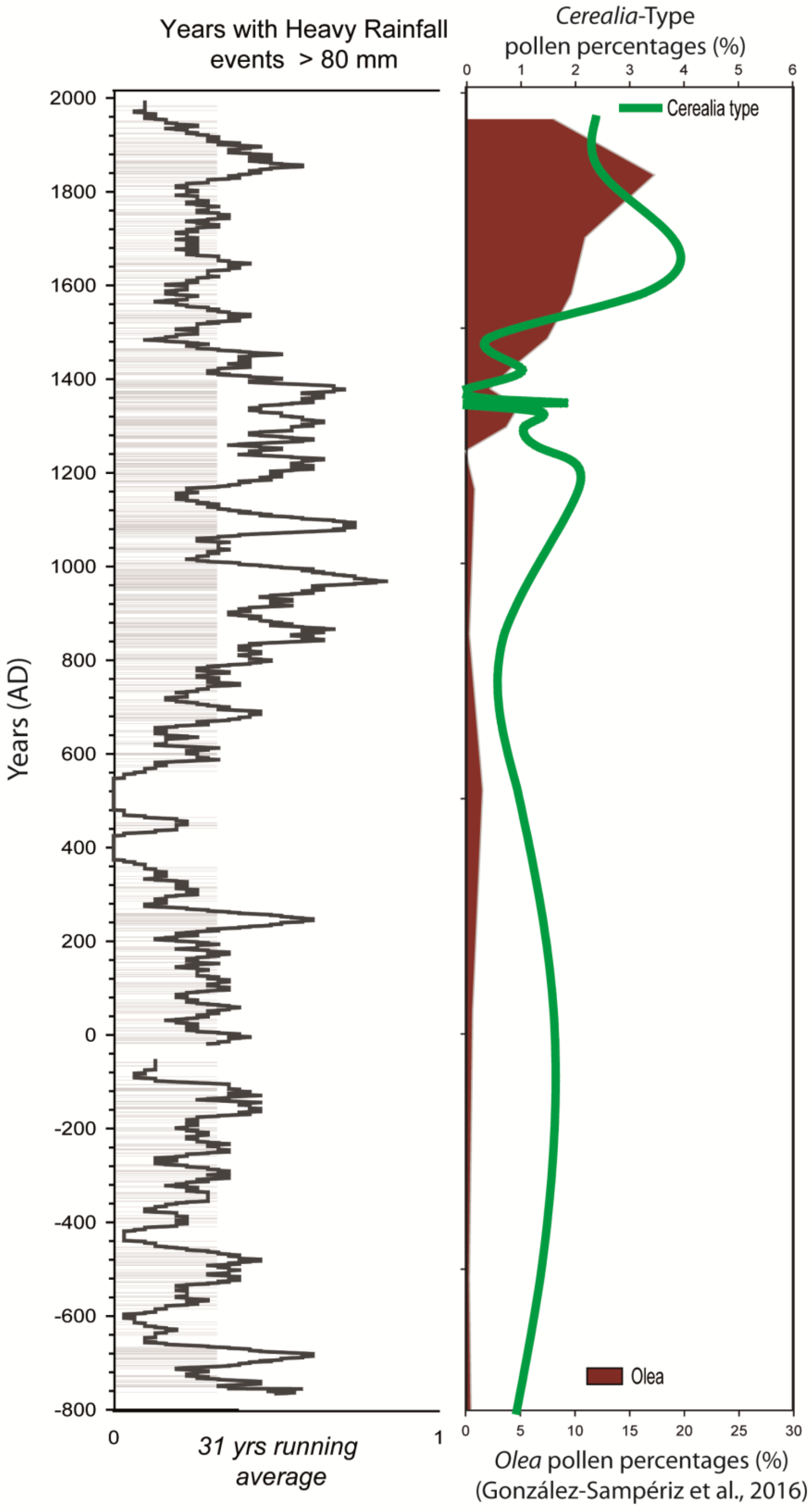

Figure S4: Comparison between total detrital layers frequency in Lake Montcortès and pollen-based anthropogenic proxies from Lake Estanya [3]

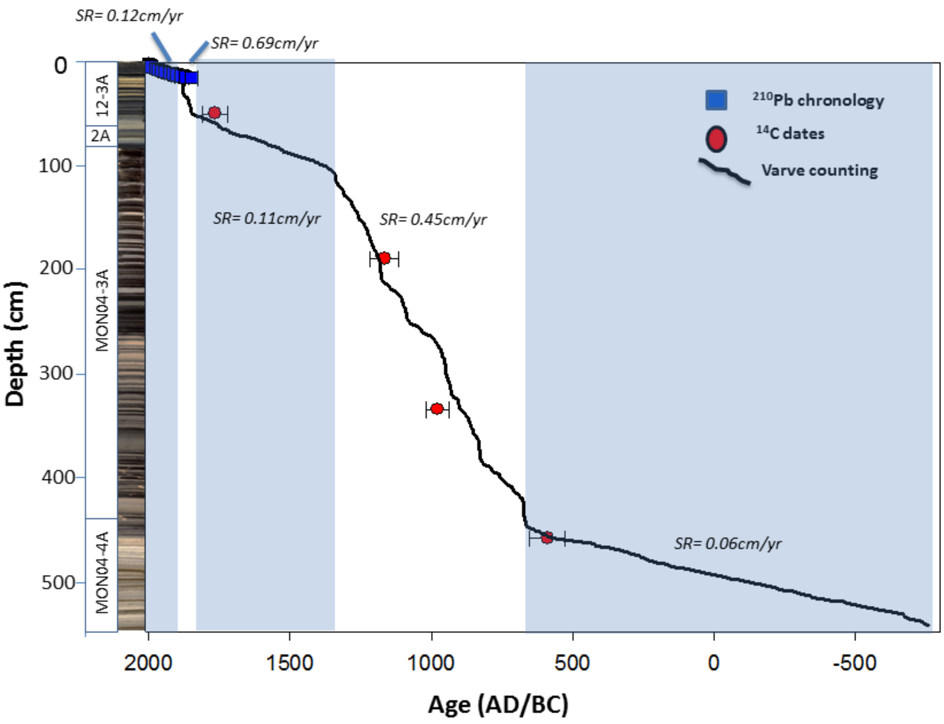

Figure S5: Composite sequence (left) and Age–depth model (right) for the last 2774 yrs (varve yr AD) in the Lake Montcortès sedimentary sequence based on varve counting. Independent radiometric chronologies are also shown -AMS ^{14}C dates 4 and ^{210}Pb [4,5]. Radiocarbon dates were recalibrated using the IntCal13 calibration curve[6].

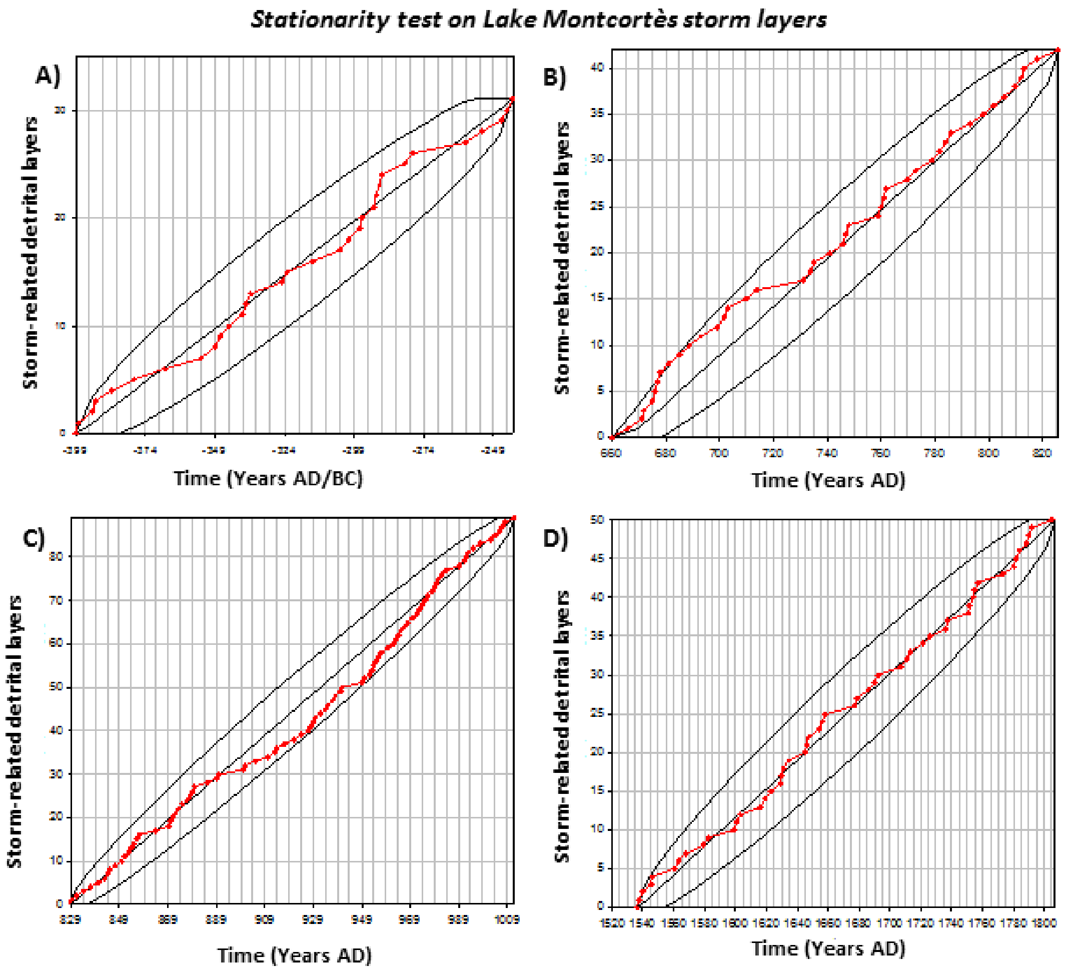

Figure S6: Poisson test on the time HR process recorded at Montcortès for selected, centennial-long periods during the IRHP, DACP, MCA and LIA (A, B, C and D respectively). The red dots show the observed accumulated number of HR recorded in the lake during the analyzed period. The central oblique line is the calculated average accumulated HR for the period, and the enveloped lines are the computed 95% tolerance interval.

TABLES

Table S1: Extreme precipitation data (Daily precipitation) from Cabdella Meteorological station for the HR >80 mm and HR >90 mm (bold), weather types, detrital layer types and the corresponding Mediterranean Oscillation index (MOi) during these dates

nº event	Date	Cold (1) /warm (2) season	Rainfall (mm)	Weather type	**Detrital layer type**	**MOi**
1	***8/9/1992***	***2***	***120***	***CYCLONIC***		***0.28***
2	*7/3/1991*	*1*	*85*	*CYCLONIC*		*-4.03*
3	***10/10/1987***	***1***	***93***	***CYCLONIC HYBRID NW***		***-1.41***
4	*22/1/1985*	*1*	*88*	*CYCLONIC*		*-1.96*
5	***9/11/1984***	***1***	***105***	***CYCLONIC***	**N-C DL**	**-2.21**
6	***7/11/1982***	***1***	***252***	***CYCLONIC***	**DL (2b)**	**-2.69**
7	*26/8/1980*	*2*	*81*	*CYCLONIC*		*0.10*
8	***25/3/1979***	***1***	***96***	***ADVECTIVE PURE SW***		***0.19***
9	***6/10/1977***	***1***	***90***	***CYCLONIC***		***-2.72***
10	***31/12/1976***	***1***	***93***	***CYCLONIC HYBRID SW***		***-1.14***
11	*28/61974*	*2*	*84*	*ADVECTIVE PURE W*	N-C DL (1)	0.00
12	*16/11/1967*	*1*	*88*	*CYCLONIC*		*-1.54*
13	*8/11/1966*	*1*	*83*	*CYCLONIC*		*-1.08*
14	*25/9/1965*	*2*	*84*	*CYCLONIC HYBRID W*		*-1.89*
15	***24/2/1964***	***1***	***95***	***CYCLONIC HYBRID SW***		***-1.05***
16	*18/4/1964*	*1*	*86*	*CYCLONIC*		*-1.04*
17	***4/10/1960***	***1***	***95***	***ADVECTIVE PURE SW***		***0.74***
18	*21-22/11/1960*	*1*	*85-83*	*CYCLONIC HYBRID SW*		*-0.72/-2.73*
19	***3/3/1959***	***1***	***103***	***CYCLONIC HYBRID SW***		***-1.37***
20	*9/12/1959*	*1*	*86*	*CYCLONIC*		*-1.47*
21	***19/12/1958***	***1***	***95***	***ADVECTIVE PURE SW***		***-1.79***
22	*22/3/1957*	*1*	*80*	*CYCLONIC HYBRID SW*		*0.27*
23	*27/8/1955*	*2*	*83.3*	*CYCLONIC*		*-1.69*
24	***28/3/1952***	***1***	***107.5***	***CYCLONIC***	**DL (2c)**	**-3.90**
25	***11/11/1950***	***1***	***112***	***ADVECTIVE PURE W***	**N-C DL (2b)**	**0.76**
26	*27/11/1947*	*1*	*80*	*CYCLONIC*	N-C DL (2b)	-2.19
27	*24/3/1945*	*1*	*80*	*ANTICYCLONIC HYBRID NW*		*-0.83*

28	***24/3/1943***	***1***	***95.5***	***ADVECTIVE PURE W***		***-1.25***
29	*29/10/1942*	*1*	*85*	*ADVECTIVE PURE W*		*-2.00*
30	*11/11/1941*	*1*	*85.5*	*CYCLONIC HYBRID NW*		*-2.97*
31	*22/6/1939*	*2*	*81*	*CYCLONIC*		*0.21*
32	***25-27/10/1937***	***1***	***160****-80-80*	***ADVECTIVE PURE W-*** *CYCLONIC*	**FT (2a)**	**-2.88/-4.63/-3.82**
33	***25/12/1935***	***1***	***101***	***ANTICYCLONIC HYBRID W***	**DL**	**-1.90/**
34	*10/3/1935*	*1*	*85*	*CYCLONIC HYBRID NW*		*-0.11*
35	*9/12/1934*	*1*	*82*	*ADVECTIVE PURE W*		*-0.58*
36	***8/11/1927***	***1***	***91.5***	***ADVECTIVE PURE WW***	FT (2a)	-2.94
37	***27-28/10/1926***	***1***	***109-****83*	***ANTICYCLONIC HYBRID SW-*** *ADVECTIVE PURE NW*		***-0.27/-2.27***
38	*18-20/11/1926*	*1*	*81-80.5*	*ADVECTIVE PURE W*		*-1.89/0.77*
39	***12/8/1924***	***2***	***120***	***CYCLONIC***	**FT (1)**	**-0.94**
40	***27/11/1923***	***1***	***94***	***CYCLONIC***	**FT (2a)**	**-1.91**

Supplementary information references

1 Pauling, A., Luterbacher, J., Casty, C. & Wanner, H. Five hundred years of gridded high-resolution precipitation reconstructions over Europe and the connection to large-scale circulation. *Climate Dynamics* **26**, 387-405 (2006).

2 Luterbacher, J. *et al.* Reconstruction of sea level pressure fields over the Eastern North Atlantic and Europe back to 1500. *Climate Dynamics* **18**, 545-561, doi:10.1007/s00382-001-0196-6 (2002).

3 González-Sampériz, P. *et al.* Environmental and climate change in the southern Central Pyrenees since the Last Glacial Maximum: A view from the lake records. *CATENA* (2016).

4 Corella, J. P. *et al.* The 1.5-ka varved record of Lake Montcortès (southern Pyrenees, NE Spain). *Quaternary Research* (2012).

5 Corella, J. P., Benito, G., Rodriguez-Lloveras, X., Brauer, A. & Valero-Garcés, B. L. Annually-resolved lake record of extreme hydro-meteorological events since AD 1347 in NE Iberian Peninsula. *Quaternary Science Reviews* **93**, 77-90, doi:http://dx.doi.org/10.1016/j.quascirev.2014.03.020 (2014).

6 Reimer, P. J. *et al.* IntCal13 and Marine13 radiocarbon age calibration curves 0-50,000 years cal BP. (2013).
